# Associations between Ethnic Minority Status and Popularity in Adolescence: the role of Ethnic Classroom Composition and Aggression

**DOI:** 10.1007/s10964-020-01200-6

**Published:** 2020-02-07

**Authors:** Gonneke W. J. M. Stevens, Carolien Veldkamp, Zeena Harakeh, Lydia Laninga-Wijnen

**Affiliations:** 1 Interdisciplinary Social Science, Utrecht University, Utrecht, The Netherlands; 2 Municipal Health Services Amsterdam, Amsterdam, The Netherlands; 3 Child Health, TNO, Leiden, The Netherlands; 4 Department of Sociology, University of Groningen, Groningen, The Netherlands

**Keywords:** Ethnic minorities, Popularity, Ethnic classroom composition, Aggression, Early adolescents

## Abstract

Although there are theoretical reasons to expect an association between ethnic minority status and popularity, research on this topic is scarce. Therefore, this association was investigated including the moderating role of the ethnic classroom composition and the mediating role of aggression. Data from the longitudinal Dutch SNARE (Social Network Analysis of Risk behavior in Early adolescence) project were used among first-year students (comparable to 5th grade) (*N* = 1134, *N*_classrooms_ = 51, *M* = 12.5 years, 137 non-Western ethnic minority students). Popularity and aggression were assessed with peer nominations. Multi-level Structural Equation Models showed that ethnic minority status was indirectly associated with higher popularity, through higher aggression. Moreover, with increasing numbers of ethnic minority students in the classroom, popularity levels of both ethnic majority and ethnic minority students decreased. Only when differences in aggression between ethnic minority and majority students were included in the analyses, while the ethnic classroom composition was not included, lower popularity levels were found for ethnic minority than ethnic majority students. Scientific and practical implications of this study were addressed in the discussion.

## Introduction

When young people reach adolescence, peer relationships gain importance. Due to changes in their social brain, adolescents become increasingly aware of their position in their peer group and motivated to pursue being noticed, approved, and powerful among their peers (Prinstein 2018; Chein et al. 2011). They prioritize popularity—a social reputation characterized by power, prestige, and admiration (Cillessen and Marks 2011)—over other social and relational goals (LaFontana and Cillessen 2010), potentially because having a popular position earns youth access to valuable social and material resources (resource control theory, Hawley 2003). Popularity can have both negative and positive consequences for adolescent development. Popular students are more likely to show low academic performance (Zhang et al. 2018) and to engage in risk behaviors compared to their agemates (e.g., Moody et al. 2011). Also, popular students have been found to have better social skills, more self-confidence, and lower levels of depression (Meijs et al. 2010; Sandstrom and Cillessen 2010).

**Fig. 1:**
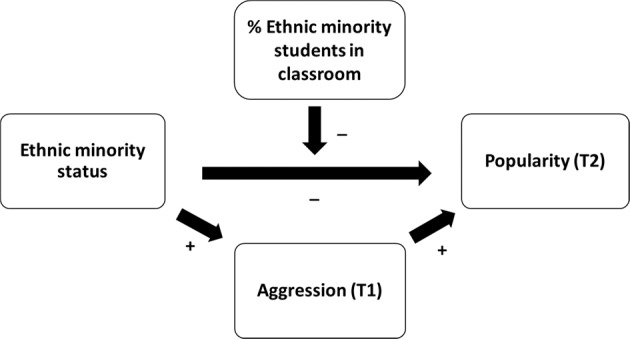
Conceptual model for the association between ethnic minority status and popularity

Although many studies have investigated the association between individual characteristics and popularity, there is a scarcity of research examining if and how ethnic minority status is related to popularity. This is unfortunate, as societies throughout the world are becoming increasingly ethnically mixed because of growing numbers of international migrants (United Nations 2017), and, as mentioned above, popularity is in several ways related to adolescent development. Also, as will be described in detail below, several theoretical perspectives provide us with what may seem opposing expectations about the impact of having an ethnic minority status on popularity. Ethnic minority status can be hypothesized to be associated with higher popularity via higher aggression. Alternatively, especially in classrooms with many ethnic majority members, ethnic minority status can be expected to be associated with lower popularity (Rock et al. 2011; see Fig. 1 for the conceptual model of the study). To test these two opposing expectations, the association between ethnic minority status and popularity was investigated in a Dutch longitudinal sample of early adolescents, including the mediating role of aggression and the moderating role of ethnic classroom composition.

### Ethnic Minority Status and Popularity: the Mediating Role of Aggression

According to Moffitt’s theory of adolescence-limited antisocial behavior (Moffitt 1993), it can be hypothesized that engagement in antisocial behavior such as aggression leads to more popularity in adolescence. She stated that early adolescents may experience a “maturity-gap” as a consequence of the fact that they are biologically, yet not socially mature, given that they are restrained from desirable adult behavior (e.g., voting, drinking) and required to obey authorities at school and at home. This theory presupposes that adolescents use aggression as a way to bridge this maturity-gap. For instance, one can prove to be socially mature by showing aggressive behavior toward peers and teachers, bullying and skipping classes (Odgers et al. 2008). Thus, aggressive students may be perceived as socially mature, which in turn increases their power and their level of popularity.

Previous research indeed found support for the positive association between aggression and popularity. Scholars showed that both relational and physical aggression predicted popularity at a later time point (Cillessen and Borch 2006; Garandeau et al. 2011; Rose et al. 2004). Yet it is also important to acknowledge that aggression may not relate to popularity for all adolescents. Indeed, research identified two types of aggressive youth, with one group containing non-socially prominent aggressors relegated to peripheral positions in the peer group and a second group containing highly central, aggressive leaders (e.g., Laninga-Wijnen et al. 2020). Given that ethnic minority adolescents may react with aggression to the discrimination they face (Verkuyten 2008), and reactive aggression less likely results into popularity than proactive aggression (Stoltz et al. 2016), aggression might not, or to a lesser extent, be associated with more popularity in the current population. Still, given the above theoretical notion and the available literature, a positive association between aggression and popularity is expected.

In addition to the potentially positive association between aggression and popularity, a positive association between ethnic minority status and aggression is expected. There are two possible reasons why students perceive ethnic minority students as more engaged in aggression than ethnic majority students. First, this perception may stem from actual behavior of ethnic minority adolescents, given that ethnic minority youth in the Netherlands reported higher levels of aggression compared to ethnic majority youth (Adriaanse et al. 2014; Duinhof et al. 2015). Second, this perception may result from existing attitudes concerning the behavior of ethnic minority youth, as ethnic minority status has been stereotypically associated with engagement in aggressive behaviors (Clemans and Graber 2016). In conclusion, due to either actual behaviors or stereotypes, ethnic minority students may be viewed as more aggressive than ethnic majority students. Combining the two expectations, it can be hypothesized that ethnic minority students are perceived as more popular than ethnic majority students because of their (alleged) higher involvement in aggression.

### Ethnic Minority Status and Popularity: the Moderating Role of Ethnic Classroom Composition

In contrast to the reasoning above, ethnic minority students may also be expected to be less popular than their ethnic majority peers, especially in classrooms with many ethnic majority students. Overall, in the Netherlands as well as in many countries throughout the globe, members of ethnic minorities have a lower social standing than members of the ethnic majority (e.g., Lee et al. 2019; Zick et al. 2008). To illustrate, ethnic minority groups are underrepresented in higher education and are more likely to be unemployed than Dutch ethnic majority members (Huijnk and Andriessen 2016). Also, ethnic minorities in the Netherlands face a considerable amount of prejudice and discrimination (Huijnk et al. 2014) and intergroup social exclusion is disproportionally experienced by children and adolescents from ethnic minority groups (e.g., Verkuyten 2008). As such, ethnic minority adolescents may be more likely to have limited power than ethnic majority adolescents. Since popularity is a social reputation characterized by power, prestige and admiration (De Bruyn and Cillessen 2006), it can be hypothesized that ethnic minority adolescents are lower in popularity than ethnic majority adolescents.

However, researchers studying peer status of ethnic minority and majority adolescents have also acknowledged the significance of the ethnic composition of the classroom (e.g., Bellmore et al. 2011). Especially in classrooms with many ethnic majority members, ethnic minority students may be less popular than ethnic majority students (Garandeau et al. 2011; Wilson and Rodkin 2011). In these classrooms, there is less opportunity for intergroup contact, which typically enhances intergroup prejudice, stigma and discrimination through processes of unfamiliarity, uncertainty and anxiety (Paluck et al. 2019; Pettigrew and Tropp 2006). In turn, this may negatively impact upon the social standing and popularity of ethnic minorities. Alternatively, especially when ethnic minority adolescents are in the numerical minority in the classroom, they may be perceived of as a “misfit” as they deviate from the (ethnic) group norm (Jackson et al. 2006; Nadeem and Graham 2005). Accordingly, especially in classes with many ethnic majority students, ethnic minority adolescents are expected to be less popular than their agemates from the ethnic majority.

### Empirical Research on Ethnic Minority Status and Popularity

Notwithstanding the characterization of Western societies as increasingly ethnically mixed and the theoretical likelihood of ethnic minority status being associated with popularity, there is a scarcity of research on this topic. The available research is US-based and indicates that in studies in which African Americans mostly were in the numerical majority in their classrooms, they are perceived as cooler and having more leadership skills than European American youth (Garandeau et al. 2011; Jackson et al. 2006; Wilson and Rodkin 2011). However, in a study in which African Americans were in the numerical minority in most classrooms, they were perceived as less popular than European American adolescents (Rock et al. 2011). These different effects according to the ethnic classroom composition may have been explained by the finding that characteristics such as “coolness” and “leadership skills” are more often ascribed to peers of the same ethnic group than to peers of a different ethnic group (Bellmore et al. 2007; Jackson et al. 2006; Jamison et al. 2015). Thus, there is a lack of empirical research on the impact of ethnic minority status on adolescent popularity, especially outside of the US and for other ethnic groups than African and European Americans. Also, research has not systematically investigated the moderating role of the ethnic classroom composition or the mediating role of aggression in this association. Still, based on the empirical literature, there is some evidence to suggest an association between ethnic minority status and popularity, with the ethnic classroom composition crucially impacting upon the direction of this association.

## Current Study

This study investigates the association of ethnic minority status and popularity among early adolescents, including the moderating effect of the ethnic classroom composition and the mediating effect of aggression, using a Dutch longitudinal study of students in their first year of secondary education (comparable to 5th grade). Because the vast majority of adolescents hardly knew anybody in the new classroom, this provides us with the opportunity to investigate the establishment and development of aggression and popularity when adolescents enter a new peer context. The following hypotheses were generated from the literature. First, ethnic minority students can be expected to be more popular than ethnic majority students, because empirical research suggests them to be higher in aggression and Moffitt’s theory of adolescence-limited delinquency assumes and several studies found a positive association between aggression and popularity. Second and potentially in contrast, because ethnic minorities have a relatively low social standing in society, ethnic minority students can also be expected to be relatively unpopular. This may be especially true whenever ethnic minority adolescents are in a classroom with high percentages of ethnic majority students, as in these school classes intergroup prejudice and discrimination may be particularly high and/or ethnic minority students may be more likely to be perceived as a “misfit”.

## Methods

### Participants

Data from the longitudinal SNARE project (see for more information Dijkstra et al. 2015; Franken et al. 2016) on adolescent social and behavioral development were used. All first-year students of two secondary schools in the Netherlands were approached to take part at the beginning of the academic year 2011–2012 (cohort 1). A new cohort of students entering first year of these two secondary schools the following academic year (2012–2013), was approached as well (cohort 2). In the Netherlands, at the start of secondary education, most adolescents know hardly anybody in their classroom (based on available information, it was estimated that fewer than two students per classroom came from the same primary school; see for more information Laninga-Wijnen et al. 2018).

For both first-year cohorts, data were collected three times in one academic year. The first wave (T1) was one month after the students entered secondary education (in October 2011 for cohort 1 and October 2012 for cohort 2), followed by a second wave in December 2011 and 2012 (T2), and a third wave in April 2012 and 2013 (T3). The study was approved by the Internal Review Board (IRB) of one of the participating universities (Utrecht University). Students received an information letter for themselves and their parents explaining the purpose of the study. Parents who did not wish their children to participate in the study were asked to indicate this, and students were made aware that they could opt out anytime. Surveys were completed under supervision of a teacher and a research assistant, who kept the students from talking or peeking at each other’s computers.

Of the 1144 first-year students that were approached, 0.9% declined to participate for various reasons (i.e., the adolescent was dyslectic or the research was perceived to be too time-consuming). This yielded a sample of 1134 first-year students from 51 classrooms; with 568 (50.1%) boys and 566 (49.9%) girls, who were on average *M* = 12.66 (SD = 0.56) years old. Each school class had 12–30 students (*M* = 22.24 students per classroom). At the end of elementary school, in sixth grade (at age 11–12), students are selected into a tracked secondary school, primarily based on a teacher assessment. In our sample, 46.7% of the students were enrolled in lower-level education (including preparatory secondary school for technical and vocational training), and 53.3% were attending higher-level education (including preparatory secondary school for higher professional education and for university). Family socioeconomic status was determined by means of families’ zip codes for which “status scores” were assessed by the Dutch Social Cultural Planning Office (Benson et al. 2015). These status scores were calculated based on the percentage of inhabitants with a low educational level and with relatively low incomes, the average income of inhabitants within an area, and the percentage of unemployed inhabitants. A small percentage of participants (4.5%) had a high socioeconomic status, 53.1% had a moderate socioeconomic status and 33.2% had a low socioeconomic status (no data were available for 9.2% of our sample). Compared to the average socioeconomic status of inhabitants in the Netherlands, our sample had a somewhat lower socioeconomic status. In total, 12.1% of the students belonged to a non-Western ethnic minority, of which 85.4% were second generation immigrants. The group of non-Western ethnic minority students was composed of the following ethnic backgrounds: Moroccan (29.9%), Surinamese (10.9%), Turkish (9.5%), Antillean (5.8%), Indonesian (5.8%) and other such as Iran, Iraq, Somalia, China, Afghanistan, India, and Vietnam (38.0%). Of all respondents, 71.7% attended the school in the North of the Netherlands. At this school, 3.6% of the students had a non-Western ethnic background. The remainder of the participants (28.3%) attended the school in the center of the Netherlands, where 35.5% of the students had a non-Western ethnic background.

### Measures

#### Peer-nominated variables

Aggression and popularity were assessed using peer nominations from classmates. Participants could select an unlimited number of same-sex and opposite-sex classmates, and there also was the option of selecting “nobody”. The latter allowed for a differentiation between missing responses and valid empty responses for a certain respondent. Names of all pupils in a classroom were presented in a random order to avoid answering tendencies. To take differences in the number of respondents per classroom into account, the number of times an individual was nominated by classmates was divided by the number of classmates who made nominations minus one (as the respondent was not able to select him- or herself), times 100. This yielded scores ranging from 0 (received no nominations) to 100 (received nominations from everyone in the classroom).

In line with many former studies (e.g., Hopmeyer Gorman et al. 2011; Laninga-Wijnen et al. 2017), peer-nominated popularity was assessed by asking participants “Who are most popular in your class?” at Time 1, Time 2, and Time 3. This question stems from a tradition where elaborate descriptions are usually not provided for the term popular, as this is a term that has an immediate meaning to adolescents and its validity may be lost or diluted when adults impose a meaning on the term (Cillessen and Marks 2011). Evidence in favor of this strategy comes from studies that have used open-ended question formats (e.g., “What makes someone popular?”) to determine what children and adolescents understand the meaning of this construct to be within their own school, cultural, or subcultural context (e.g., Xie et al. 2006).

Peer-nominated aggression (see also Logis et al. 2013; Molano et al. 2013), was assessed with five items at Time 1, Time 2 and Time 3: “Who make fun of others?”, “Who are often rude to teachers?”, “Who are picking a fight with you?”, “Who gossip about you?”, and “Who bully you?” (based on Lease et al. 2002; see also Laninga-Wijnen et al. 2017, 2018). As such, aggression was assessed as a unified construct, without consideration for its different forms (i.e., physical vs. relational) and functions (i.e., reactive vs. proactive). Most of our items assessed relational forms of aggression and one item assessed aggression toward others, whereas the other items were about aggression directed toward the nominator. Factor analyses of these five items were conducted in Mplus (version 7.0, Muthén and Muthén 1998–2012). Factor analyses for aggression at T1 were reported as this was the main mediating variable; results of factor analyses for aggression at T2 and T3 were highly similar and are available upon request. As the responses to the items were all rather skewed, the MLR estimation was applied, which employs maximum likelihood estimation for non-normal data. Models were compared using the Satorra-Bentler (SB) scaled chi-square difference test (Satorra and Bentler 2010). After freeing covariance between the items “who makes fun of others?” and “who are often rude to teachers?”, the confirmatory factor analysis showed a good model fit (SB *χ*^2^(4) = 12.617, *p* = 0.013, RMSEA = 0.044, and CFI = 0.989). The standardized factor loadings ranged from 0.58 to 0.79. A model constraining the factor loadings on the individual and the classroom level to be equal, showed an acceptable to good fit (SB *χ*^2^(12) = 52.743, *p* < 0.001, RMSEA = 0.055, and CFI = 0.956), indicating the factor structure on the individual level and the classroom level to be similar. Finally, on the individual level, a test for measurement invariance between ethnic minority and ethnic majority students was conducted. The model with scalar invariance showed a good fit (SB *χ*^2^(16) = 28.237, *p* < 0.05, RMSEA = 0.037, and CFI = 0.987), indicating that aggression could be measured by the same items for both ethnic minority and majority students.

#### Ethnic minority status

Participants were asked in which country their father and their mother was born, to generate a dichotomous variable contrasting non-Western ethnic minority students (1) with all other students (i.e., including students with a Western ethnic background other than the Netherlands) (0). Students who had their origin in Asia, South-America, Africa, Turkey or Indonesia were assigned as having a non-Western ethnic background. Non-western immigrants constitute by far the largest group of ethnic minority adolescents in the Netherlands. Adolescents with a Western ethnic background were not included in the former group, as non-western and western immigrant adolescents differ considerably in their family’s socioeconomic status (e.g., Netherlands Inspectorate of Education 2018), and socio-cultural distance between the origin and receiving country (Kalmijn 2015). In line with the definition of the Dutch Central Statistics Office, a student was considered as having another ethnic background than Dutch if one or both of his/her parents were born outside the Netherlands. If both parents were born abroad and in different countries (which was only true for 3% of our ethnic minority students), the student was assigned the ethnicity of the mother, because of the notion that familial cultural socialization of adolescents is mostly influenced by their mothers (e.g., Knight et al. 2011).

#### Classroom percentage of ethnic minority students

For each classroom, the percentage of ethnic minority students was determined based on the percentage of non-Western ethnic minority students in the classroom at Time 1. This assessment was adequate for the whole year, as only 11 students changed classrooms during the year. Students with missing data on the variable ethnic minority status (2.8%), were not included in the calculation. The percentage of ethnic minority students in the classrooms ranged from 0 to 70.0%, with a mean of 12.0% (SD *=* 16.4). The (moderating) effects of the ethnic classroom (instead of the school) composition were investigated, as in the Netherlands students are in the same classroom every day, all day long, across the whole school year.

#### Control variables

Sex, age, and adolescent education were included as control variables. Age was included as a continuous variable, centered around the mean, and measured by the specific date of birth. Education had six categories, ranging from three levels of pre-vocational education (1–3), general secondary education (4), a combination of secondary and pre-university education (5) to pre-university education (6).

### Analytic Strategy

To test the main model, multi-level structural equation modeling (SEM) was performed in Mplus. There were 33 adolescents with missing data (mostly on ethnic minority status) and these were therefore excluded from the analyses in Mplus. Attrition analyses showed no significant differences between adolescents with missing data on ethnic minority status and complete cases. In the main analyses, data were used from T1 (ethnic minority status, sex, age, adolescent education, aggression, ethnic classroom composition) and T2 (popularity). Longitudinal data were particularly relevant for testing the indirect effect from ethnic minority status to popularity through aggression. Regarding the direct linkage between ethnic minority status and popularity and the (moderating) role of the ethnic classroom composition, the main interest was in predicting popularity, rather than in predicting changes in popularity from the first to second wave. This resulted in the following analytical steps.

To start, variances at the individual and the classroom level were examined. In the first model, the direct effect of ethnic minority status on popularity at Time 2 was tested, while including all control variables (sex, age, and adolescent education). In the second model, aggression at Time 1 was included as predictor of popularity at Time 2. The indirect effect of ethnic minority status on popularity at Time 2 via aggression at Time 1 was tested with and without controlling for popularity at Time 1. In the third model, the variable ethnic classroom composition was included at the between-level of our model. The direct effect of this classroom-level variable on popularity at Time 2 was investigated, followed by a fourth model including the cross-level interaction between the ethnic classroom composition and ethnic minority status on popularity at Time 2. Moreover, in additional analyses, the robustness of the findings was investigated, by testing the same models for popularity at Time 1 and popularity at Time 3. All models were fitted with the MLR estimator.

## Results

### Descriptive Statistics

In Table 1, differences in perceived aggression and popularity (Time 1, Time 2, Time 3) between ethnic minority and ethnic majority students are presented. No differences in popularity were found at Time 1, while ethnic minority students showed lower scores on popularity than ethnic majority students at Time 2 and Time 3. Repeated measures analyses indicated an interaction between ethnic minority status and time on popularity (*F*_greenhouse-Geiser_(1.897) = 4.971, *p* = 0.008), implying that ethnic majority students became more popular over the course of the year, while ethnic minority students became less popular during the same period. Also, ethnic minority students were scored higher on perceived aggression than ethnic majority students. In Table 2, correlations between popularity and aggression are shown separately for ethnic minority and majority students. Popularity throughout the year was positively related to perceived aggression at Time 1 for both groups of students.

**Table 1:** Comparison of mean levels of popularity and aggression between ethnic minority (*n* = 137) and ethnic majority students (*n* = 965)

	Total	Ethnic Majority	Ethnic Minority	
	Mean (SD)	Mean (SD)	Mean (SD)	*t*
Popularity T1	13.78 (15.40)	13.81 (15.57)	13.57 (14.22)	0.183
Popularity T2	14.97 (16.84)	15.35 (17.23)	12.36 (13.60)	2.319*
Popularity T3	14.87 (17.75)	15.26 (18.26)	12.08 (13.41)	2.470*
Aggression T1	3.60 (5.42)	3.45 (5.33)	4.66 (5.98)	−2.452*

Nesting of students into classrooms was not taken into account

*p< 0.05

**Table 2:** Correlations between popularity and aggression, for ethnic minority students (below the diagonal) and ethnic majority students (above the diagonal)

Variable	Pop T1	Pop T2	Pop T3	Aggr T1
1. Popularity T1	–	0.782***	0.731***	0.426***
2. Popularity T2	0.725***	–	0.825***	0.421***
3. Popularity T3	0.621***	0.713***	–	0.369***
4. Aggression T1	0.450***	0.361***	0.331***	–

Nesting of students into classrooms was not taken into account

*Pop* Popularity, *Aggr* Aggression

***p < 0.001

### Associations between Ethnic Minority Status and Popularity at Time 2

Variance in popularity at Time 2 at the individual level and classroom level was assessed. The model with a random intercept for popularity at Time 2 at the classroom level had a better fit than the model with a fixed intercept (Satorra-Bentler Δ*χ*^2^(1) = 15.36, *p* < 0.001, ΔAIC = 15.5). This indicates that there was variance in popularity at Time 2 at the classroom level. The variance in popularity at Time 2 was 267.89 at the individual level, and 14.39 at the classroom level. The Intra Class Correlation was 0.047, which means that 4.7% of the variance in popularity could be explained at the classroom level.

Next, the structural equation model was tested. This model was built up step by step, resulting in four different models, presented in Table 3. In Model 1, the direct effect of ethnic minority status on popularity at Time 2 was tested, while controlling for sex, age, and adolescent education. The results showed no direct effect of ethnic minority status on popularity Time 2. In Model 2, aggression was added to the model, which means that effects of ethnic minority status on popularity at Time 2 were tested, now controlling for aggression T1, sex, age and adolescent education (Model 2, direct effects on popularity T2). In this model, ethnic minority students showed lower scores on popularity than ethnic majority students, and aggression at Time 1 had a positive effect on popularity at Time 2. Also, ethnic minority students were perceived by their classmates as more aggressive than ethnic majority students at Time 1 (Model 2, effects on aggression T1), while controlling for sex, age and adolescent education. The indirect effect of ethnic minority status on popularity at Time 2 via aggression at Time 1 was positive and significant (Model 2, indirect effect on popularity T2). This indirect effect was still significant after controlling for popularity at Time 1 (*b* = 0.348, *p* = 0.025; not reported in Table 3), indicating that aggression had a positive effect on popularity at Time 2, irrespective of popularity at Time 1. In sum, ethnic minority status was directly associated with *lower* levels of popularity when controlling for aggression, and indirectly with *higher* levels of popularity through higher levels of aggression.

**Table 3:** Unstandardized and standardized coefficients for the models on ethnic minority status and popularity at Time 2

	Model 1		Model 2		Model 3		Model 4
	*B*	Beta	*B*	Beta	*B*	Beta	*B*
Direct effects on popularity T2							
Individual level							
Ethnic minority status	−1.928	−0.039	−2.758*	−0.055	−1.085	−0.022	−0.938
Sex (boy)	2.313**	0.070	−0.274	−0.008	−0.268	−0.008	−0.250
Age	3.155**	0.096	3.056**	0.093	3.084*	0.094	3.053*
Adolescent education	−0.434	−0.038	0.327	0.029	0.328	0.029	0.331
Aggression T1			3.049***	0.428	3.060***	0.429	3.051***
Classroom level							
% minority students in classroom					−0.159***	−0.579	−0.169***
% minority students in classroom × ethnic minority status							0.017
Effects on aggression T1							
Ethnic minority status			0.548*	0.078	0.549*	0.078	0.543*
Sex (boy)			0.888***	0.192	0.887***	0.192	0.887***
Age			0.094	0.020	0.093	0.020	0.093
Adolescent education			−0.201*	−0.126	−0.203*	−0.127	−0.203**
Indirect effect on popularity T2							
Ethnic minority status via aggression T1			1.672*	0.034	1.680*	0.034	–
Residual variances							
Intercept: student level	265.46***		218.74***		218.60***		218.71***
Intercept: classroom level	13.16**		17.63***		12.24***		12.00***
Slope of ethnic min. status							−0.938
Covariance slope and intercept							−0.145

*p< 0.05; **p< 0.01; ***p< 0.001

In Model 3, the ethnic classroom composition was included at the classrooms level (Model 3, direct effect on popularity T2). In classrooms with higher percentages of ethnic minority students, popularity scores at Time 2 were lower. The negative direct effect of ethnic minority status on individual-level popularity at Time 2, which was found in Model 2, disappeared when controlling for the ethnic classroom composition (Model 3, direct effects on popularity T2). The positive indirect effect of ethnic minority status on popularity at Time 2 via a higher level of aggression at Time 1 remained significant (Model 3, indirect effect on popularity T2), which was also true for this effect when controlling for popularity at Time 1 (*b=* 0.352, *p* = 0.026; not reported in Table 3).

**Fig. 2:**
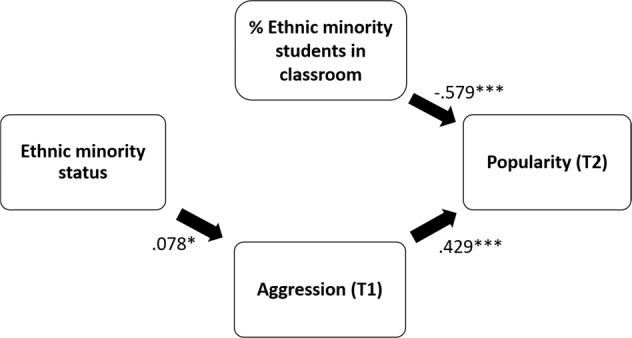
Final model with standardized coefficients for the association between ethnic minority status and popularity. In our model we controlled for sex, age and adolescent education. *p < 0.05; ***p < 0.001

In Model 4, a cross-level interaction was included, specifying the ethnic classroom composition as a moderator on the direct relation between ethnic minority status and popularity (Model 4, direct effects on popularity T2). The slope of ethnic minority status on popularity at Time 2 did not vary between classrooms, and no interaction was found between ethnic classroom composition and ethnic minority membership on popularity. This means that the effect of ethnic minority status on popularity at Time 2 did not vary with the classroom studied, and more specifically that this effect did not vary with the percentage of ethnic minority students in the classroom. Because of the insignificant interaction included in Model 4, Model 3 can be seen as the final model and is presented in Fig. 2 [RMSEA = 0.071; CFI = 0.869; SRMR_within_ = 0.052; SRMR_between_ = 0.051]. It shows that ethnic minority students are more likely to be perceived as aggressive by their classmates, which in turn was associated with higher levels of popularity. Additionally, with increasing numbers of ethnic minority students in classrooms, decreasing mean levels of overall popularity are found.

### Additional Analyses

In order to test the robustness of the findings, the same models were tested for popularity at Time 1 and popularity at Time 3. Overall, results were highly similar to the findings for popularity at Time 2, and are available upon request. In contrast to popularity at Time 2, for popularity at Time 1, no direct negative effect of ethnic minority status on popularity was found when controlling for aggression at Time 1. Similar to popularity at Time 2, an indirect positive association between ethnic minority status and popularity at Time 1 was found via higher levels of aggression at Time 1 (*b* = 1.597, *p* = 0.028). Also, the percentage of ethnic minority students in the classroom was negatively associated with popularity at Time 1 (*b* = −0.588, *p* < 0.001) and no interaction between ethnic minority status and the ethnic classroom composition was found.

The same four models were also tested for popularity at Time 3, including aggression at Time 2 in the model. In line with the findings for popularity at Time 2, a direct negative effect of ethnic minority status on popularity was found after controlling for aggression at Time 2 (*b* = −3.151, *p* = 0.022). Also, an indirect positive association between ethnic minority status and popularity at Time 3 was found via higher levels of aggression at Time 2 (*b* = 2.077, *p* = 0.042). Yet, this indirect effect was not significant after controlling for popularity at Time 2. Finally, higher percentages of ethnic minority students in the classroom were associated with lower popularity at Time 3 (*b* = −0.697, *p* < 0.001), and the negative direct effect of ethnic minority status on popularity Time 3 disappeared when including ethnic classroom composition. No interaction between ethnic minority status and ethnic classroom composition was found.

## Discussion

Notwithstanding the significance of popularity during adolescence and the fact that current societies are becoming increasingly ethnically mixed, there is a scarcity of research examining if and how ethnic minority status is related to popularity. Therefore, the current study examined the association between ethnic minority status and popularity, including the mediating role of aggression and the moderating role of the ethnic classroom composition, in a longitudinal sample of first year high-school students in the Netherlands. In general, the results suggest that the linkage between ethnic minority status and popularity should be seen as a combination of different processes, with aggression and the ethnic classroom composition playing an important role. More specifically, results revealed that ethnic minority status was indirectly associated with higher levels of popularity through higher levels of aggression. The results also showed that with increasing numbers of ethnic minority students in the classroom, mean popularity levels of both ethnic majority and ethnic minority members decreased. Only when differences in aggression between ethnic minority and majority students were included in the analyses, while the ethnic classroom composition was not included, lower popularity levels were found for ethnic minority than ethnic majority students.

This study is one of the first to find support for the idea that ethnic minority students show more aggression compared to ethnic majority students, and this in turn increases their level of popularity. These results are in line with former Dutch studies showing an association between ethnic minority status and aggression (Adriaanse et al. 2014; Duinhof et al. 2015). Also, it confirms Moffitt’s theory of adolescence-limited antisocial behavior (Moffitt 1993) and research revealing a positive link between aggression and popularity (Cillessen and Borch 2006; Garandeau et al. 2011; Rose et al. 2004). We did not find evidence for the side-note made in the introduction, that aggression might not be that strongly associated with popularity in our sample, since ethnic minority adolescents may react with aggression to discrimination, and reactive aggression is less strongly associated with popularity than proactive aggression (Stoltz et al. 2016). Specifically, our descriptive analyses showed similar correlations between aggression and popularity for ethnic minority and majority students. All in all, this study suggests that in order to understand the linkage of ethnic minority status and popularity, aggression needs to be taken into account.

For both ethnic minority and majority students, higher percentages of ethnic minority students in the classroom were associated with lower popularity. This finding is in contrast with our expectation that especially in classrooms with higher percentages of ethnic majority students, ethnic minority students are less popular than ethnic majority students. Also, the finding is not in line with earlier US research among African American and European youth in which it was found that African American youth are more popular, cooler or are perceived as having more leadership skills than European American youth when they are in the numerical majority in their classroom (Garandeau et al. 2011; Jackson et al. 2006; Wilson and Rodkin 2011), while the opposite was true when they were in the numerical minority (Rock et al. 2011). One explanation for this finding could be that in the Netherlands, classrooms with a high share of ethnic minorities often consist of adolescents with a great variety of ethnic backgrounds (Herweijer 2011), which is also obvious from our sample. In these classrooms, it may be rather unclear for both ethnic minority and majority adolescents which behaviors are regarded as “popular”, because behavior associated with popularity by one ethnic or cultural group, may differ from behavior associated with popularity by another ethnic or cultural group (Niu et al. 2016). Because of this unclarity, in these classrooms relatively few (ethnic minority and majority) adolescents may be nominated as popular. Additionally, according to the constrict theory (Putnam 2007), the integrative threat theory (Stephan and Stephan 2000) and former empirical research (e.g., Vervoort et al. 2011), in a context with high ethnic diversity, there may be more feelings of threat and competition, more conflict and social isolation, and less trust in both one’s own and other ethnic groups. In such climates, adolescents may be less likely to perceive ethnic minority and majority classmates as popular.

Only when including aggression and not including the ethnic classroom composition, a direct effect between ethnic minority status and popularity was found, with ethnic minority students being perceived of as less popular than ethnic majority students. This result suggests that because ethnic minority students had more chance of being in classrooms with a relatively high percentage of ethnic minority students and these classrooms were characterized by lower levels of popularity, ethnic minority students were lower in popularity than ethnic majority students. Thus the expectation formulated in the introduction that ethnic minorities in the Netherlands have a relatively low social standing which may decrease the popularity of ethnic minority adolescents in the classroom, does not seem to hold. Potentially, this generally low social standing in society does not impact upon adolescent popularity in the classroom, as for adolescents, influences on the more distant society level are less important than the more proximal classroom level (Bronfenbrenner and Morris 2007). Put differently, behaviors in the classroom may primarily influence the popularity of an adolescent, and not so much the general social status of ethnic minorities in Dutch society.

One additional finding worth mentioning, is that ethnic majority students became more popular over the course of the year, while ethnic minority students became less popular during the same period. This finding is in line with a recent study, showing decreasing patterns of coolness over the course of two years for several ethnic minority adolescents in the US, but not for the ethnic majority (Yun and Graham 2019). Considering the other results of our study, one tentative explanation for this finding could be that the social climate of ethnically mixed school classes deteriorates during the schoolyear, because competition and conflict might need some time to develop. As ethnic minority adolescents per definition are overrepresented in these classes, they may become less popular over time.

This study is one of the first to investigate the association between ethnic minority status and popularity using a longitudinal design as well as studying the moderating role of the ethnic classroom composition and the mediating role of aggression in this association. Some limitations of this study should be noted. Because the sample only contained a small number of ethnic minority students, our study did not enable a distinction between different ethnic minority groups. Therefore, possible differences in popularity between ethnic minority groups could not be tested, and the same accounted for the extent to which effects on popularity were dependent on the percentage of ethnic in-group members in the classroom. Instead, all non-Western ethnic minorities were combined in one group. Although ethnic minority groups in the Netherlands on average have a lower social status than members of the ethnic majority, ethnic hierarchy and discrimination research also showed variation in social status between ethnic minority groups (Andriessen et al. 2014; Stupar 2014). As such, while our results represent an “average” popularity across ethnic minority adolescents in the Netherlands, levels of popularity might differ according to specific ethnic minority population. Similarly, on the class level, ethnic classroom composition was assessed by means of the percentage of non-Western ethnic minority students in the classroom, instead of on percentages of co-ethnics. As research suggests that youth more often perceive same ethnic group peers as popular than peers from a different ethnic group (Bellmore et al. 2007; Jamison et al. 2015), this may have impacted the findings for the (moderating) effect of the ethnic classroom composition.

Furthermore, it could not be ruled out that the effect of the ethnic classroom composition on popularity was due to potential school-related effects. For instance, there may be school-level differences in popularity due to variation in for instance school size or social cohesion. No such information was available for our two schools. However, including school as a variable simultaneously with the ethnic classroom composition was not possible due to the high correlation between the ethnic classroom composition and the school variable (*r=* 0.84, *p* < 0.001), which would cause multicollinearity. Neither could analyses be conducted for each school separately, due to either too few ethnic minority students in school in the North of the Netherlands (28 school classes containing about 30 students with an ethnic minority background), or too few school classes in the school in the center of The Netherlands (12 classes).

Next, short intervals were used between the different timepoints, especially between T1 and T2 (2 months), which could have impacted upon the findings. Particularly, the association between popularity (T2) and aggression (T1) would likely have been weaker if longer intervals between these two assessments had been used. Finally, an aggression scale was used that combined items assessing aggression directed toward others and the nominator. Because adolescents might be less likely to indicate that they were bullied themselves than that others were bullied, some aggressive students may not have been nominated, even if they engaged in aggressive behaviors. However, previous studies have also shown that youth generally tend to overestimate their peers’ antisocial behavior toward others (Prinstein and Wang 2005), particularly the antisocial behaviors of popular peers (Helms et al. 2014). Also, many aggressive acts such as bullying occur in private (e.g., see Olweus 2013), and thus may be best assessed by asking about self-directed aggression. Moreover, all aggression items loaded strongly on one factor and the scale that was created was reliable across all waves. Therefore, this measure of aggression is expected to adequately capture aggression in the classroom context.

This study has several scientific and practical implications. Considering the scarcity of research on ethnic minority status and popularity and the limitations of the current study, future research is warranted that attempts to replicate our findings. In doing so, these studies need to distinguish between ethnic minorities originating from different ethnic backgrounds and include more secondary schools. Moreover, many research questions can be addressed that help to gain more insight into the link between ethnic minority status and popularity. Particularly, as perceptions of ethnicity-based discrimination are higher among boys than girls (Bucchianeri et al. 2016) and it is particularly the ethnic minority boys who are expected to be high in aggression (Clemans and Graber 2016), the association between ethnic minority status and popularity can be expected to be different for boys than for girls. Also, considering recent findings that being perceived of as unpopular by same-ethnicity peers may have more detrimental consequences than such perceptions by cross-ethnicity peers (Mali et al. 2019), it may be worthwhile investigating who is nominating whom. Finally, more research is warranted on the dynamics within ethnically mixed classrooms: how to understand the association between the ethnic classroom composition and popularity? Practically, the results suggest that the linkage between ethnic minority status and higher aggression, which is mostly seen as a negative phenomenon, can also have beneficial effects as popularity is for instance linked to more self-confidence and lower depression (Meijs et al. 2010; Sandstrom and Cillessen 2010). Moreover, results point to more attention for students in ethnically mixed school classes from teachers, school leaders and policymakers. Especially in these school classes, professionals may need to invest more in building up a sense of classroom unity and safety which goes across ethnic boundaries.

## Conclusion

Research examining if and how ethnic minority status is related to popularity is scarce. Therefore, the current study examined the association between ethnic minority status and popularity, including the mediating role of aggression and the moderating role of the ethnic classroom composition, in a Dutch longitudinal sample of high-school students. The most prominent results of the study included the indirect association between ethnic minority status and higher levels of popularity through higher levels of aggression. In addition, it was found that with increasing numbers of ethnic minority students in the classroom, popularity levels of both ethnic majority and ethnic minority members decreased. As such, this study may be seen as one of the initial (European) studies to unravel the linkage between ethnic minority status and adolescent’s highly valued goal of popularity.
